# Investigation of geographic disparities of diabetes-related hospitalizations in Florida using flexible spatial scan statistics: An ecological study

**DOI:** 10.1371/journal.pone.0298182

**Published:** 2024-06-04

**Authors:** Jennifer Lord, Agricola Odoi

**Affiliations:** Department of Biomedical and Diagnostic Sciences, College of Veterinary Medicine, The University of Tennessee, Knoxville, Tennessee, United States of America; University of Hong Kong, HONG KONG

## Abstract

**Background:**

Hospitalizations due to diabetes complications are potentially preventable with effective management of the condition in the outpatient setting. Diabetes-related hospitalization (DRH) rates can provide valuable information about access, utilization, and efficacy of healthcare services. However, little is known about the local geographic distribution of DRH rates in Florida. Therefore, the objectives of this study were to investigate the geographic distribution of DRH rates at the ZIP code tabulation area (ZCTA) level in Florida, identify significant local clusters of high hospitalization rates, and describe characteristics of ZCTAs within the observed spatial clusters.

**Methods:**

Hospital discharge data from 2016 to 2019 were obtained from the Florida Agency for Health Care Administration through a Data Use Agreement with the Florida Department of Health. Raw and spatial empirical Bayes smoothed DRH rates were computed at the ZCTA level. High-rate DRH clusters were identified using Tango’s flexible spatial scan statistic. Choropleth maps were used to display smoothed DRH rates and significant high-rate spatial clusters. Demographic, socioeconomic, and healthcare-related characteristics of cluster and non-cluster ZCTAs were compared using the Wilcoxon rank sum test for continuous variables and Chi-square test for categorical variables.

**Results:**

There was a total of 554,133 diabetes-related hospitalizations during the study period. The statewide DRH rate was 8.5 per 1,000 person-years, but smoothed rates at the ZCTA level ranged from 0 to 101.9. A total of 24 significant high-rate spatial clusters were identified. High-rate clusters had a higher percentage of rural ZCTAs (60.9%) than non-cluster ZCTAs (41.8%). The median percent of non-Hispanic Black residents was significantly (*p* < 0.0001) higher in cluster ZCTAs than in non-cluster ZCTAs. Populations of cluster ZCTAs also had significantly (*p* < 0.0001) lower median income and educational attainment, and higher levels of unemployment and poverty compared to the rest of the state. In addition, median percent of the population with health insurance coverage and number of primary care physicians per capita were significantly (*p* < 0.0001) lower in cluster ZCTAs than in non-cluster ZCTAs.

**Conclusions:**

This study identified geographic disparities of DRH rates at the ZCTA level in Florida. The identification of high-rate DRH clusters provides useful information to guide resource allocation such that communities with the highest burdens are prioritized to reduce the observed disparities. Future research will investigate determinants of hospitalization rates to inform public health planning, resource allocation and interventions.

## Introduction

Diabetes mellitus, a condition that affects an estimated 37.1 million adults in the United States [1], is characterized by hyperglycemia due to insulin resistance or impaired insulin production [2]. Hyperglycemia in patients with undiagnosed or inadequately controlled diabetes promotes vascular changes, which can lead to long-term complications that account for much of the morbidity and mortality associated with the condition [3]. These include accelerated cardiovascular disease, cerebrovascular disease, peripheral arterial disease, retinopathy, nephropathy and neuropathy [3, 4]. Prompt diagnosis, glycemic control, and management of cardiovascular risk factors such as hypertension and dyslipidemia are important to limit the development of these complications [5–7].

In general, hospitalizations due to diabetes complications are considered to be preventable if the condition is managed effectively in the outpatient setting [8]. However, optimal control of diabetes requires tailored patient care and relatively frequent engagement with the healthcare system, and can be challenging due to financial costs and potentially complex self-management requirements [6, 7, 9]. Thus, surveillance of diabetes-related hospitalization rates can yield important insights with respect to access, utilization, and efficacy of healthcare services [8]. Nationwide reductions in the risks of diabetes-related complications and mortality over the past few decades have been attributed to improvements in clinical care, patient education, and support [10, 11]. However, some recent reports suggest stagnation or reversal of this progress [12–14], warranting continued careful attention to this problem.

In Florida, the age-adjusted rate of hospitalizations due to diabetes increased by 19% between 2013 and 2018 [15]. During this time period, the annual cost of these hospitalizations rose from $1.8 billion to $3.6 billion [15]. It is essential to curb these trends to reduce the public health and economic impacts of diabetes, which affects over 2.4 million adults in Florida [15]. Rates of inpatient hospitalizations due to diabetes provide an indirect measure that can be used to effectively identify geographic hot-spots of poor glycemic control in the absence of a population-level hemoglobin A1c (HbA1c) registry [16]. This suggests that populations with high hospitalization rates are likely to benefit from interventions to improve diabetes management [16]. Therefore, understanding the local geographic distribution of diabetes-related hospitalization rates is important so that communities with high burdens can be prioritized for resource allocation and implementation of such interventions. However, there is a lack of information regarding the distribution of diabetes-related outcomes in Florida. Furthermore, while county-level geographic disparities in pre-diabetes prevalence, diabetes prevalence, and participation in validated self-management education and support programs have been identified within the state [17–20], information at the sub-county level is limited to model-based estimates [21] or focused, single-county investigations [22]. Filling this knowledge gap by characterizing the local distribution of diabetes-related hospitalization rates across the state will be essential to reduce disparities of adverse diabetes outcomes. Therefore, the objectives of this study were to investigate the geographic distribution of diabetes-related hospitalization rates at the ZIP code tabulation area (ZCTA) level in Florida, identify significant local clusters of high hospitalization rates, and describe characteristics of ZCTAs within the observed spatial clusters.

## Methods

### Ethics approval

This study was approved by the University of Tennessee Institutional Review Board (Number: UTK IRB-22-07182-XP). The study used secondary data collected between January 1, 2016 and December 31, 2019 and provided to the investigators by Florida Agency for Health Care Administration through a Data Use Agreement with the Florida Department of Health. The study was conducted between 2022 and 2023. Study investigators did not have information that could be used to identify individual participants during or after data collection. In accord with 45 CFR 46.116(f), informed consent was waived by the Institutional Review Board. In addition, the request for waiver of HIPAA authorization for the conduct of the study itself was approved. All data were fully anonymized before sharing with study investigators.

### Study area and design

**Fig 1:**
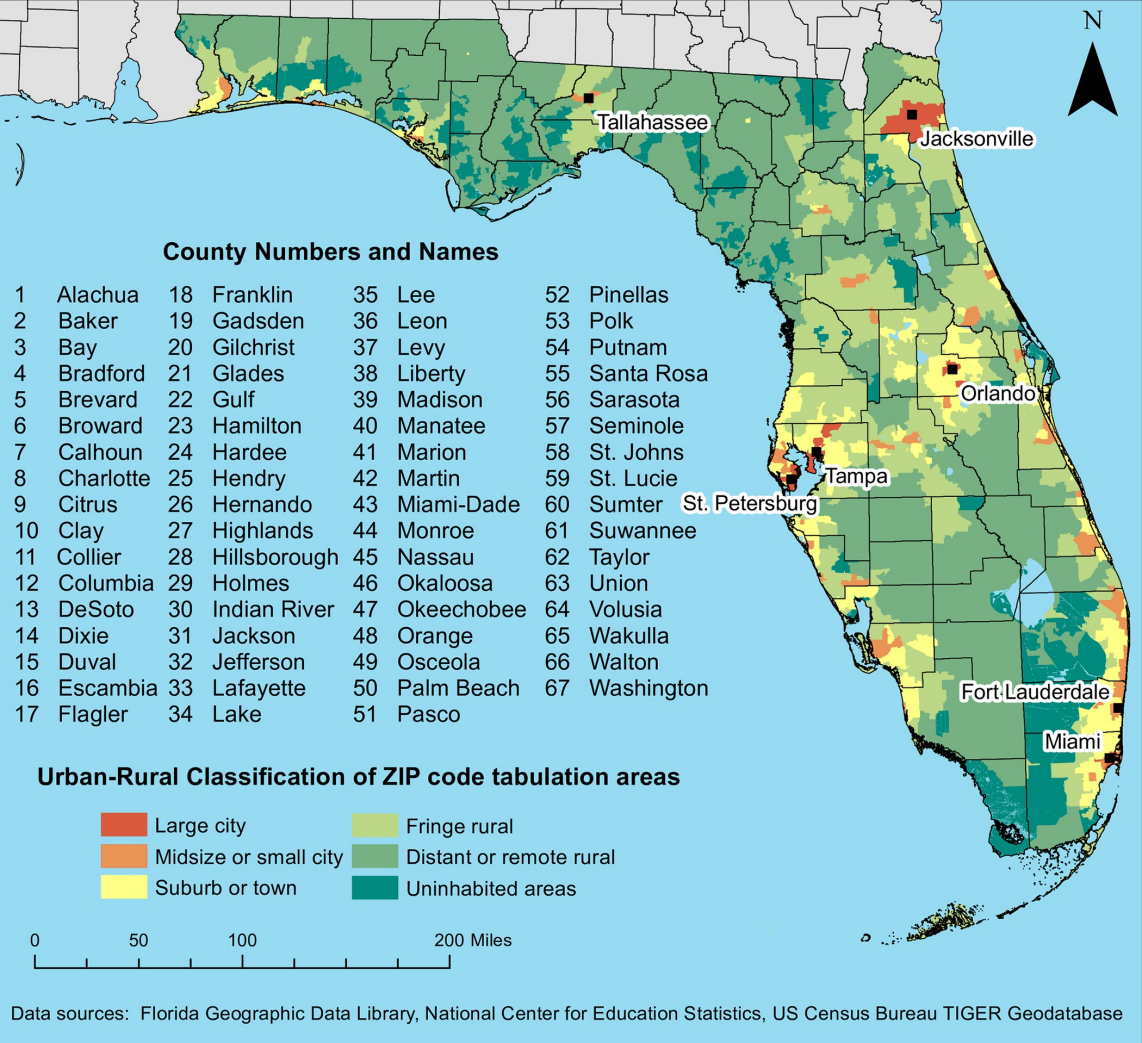
Urban-rural classification of ZIP code tabulation areas and geographic distribution of counties and major cities in Florida, USA. Figure is not copyrighted and was created by study authors using non-proprietary open-source software, QGIS.

This study was conducted in Florida, which had an estimated population of 20.9 million in 2019, the largest among the states of the Southeastern United States [23]. There are 67 counties and 983 ZIP code tabulation areas (ZCTAs) in the state (Fig 1) [24]. Approximately half (46.6%) of these ZCTAs are classified as rural and had a combined population of 6.2 million residents [23, 25]. The median age in Florida was 42 years, and the state had the highest percentage of adults aged 65 and older in the U.S. (20.1%) [23]. The estimated prevalence of diagnosed diabetes among adults in Florida in 2019 was 11.7%, but was higher among older adults, reaching 22.5% among those between the ages of 65 and 74, and 24.8% among those aged 75 and older [26]. A retrospective ecological study design was used to investigate the local geographic distribution of diabetes-related hospitalizations in Florida. Diabetes-related hospitalizations were computed at the ZCTA level, and significant high-rate spatial clusters were identified. Descriptive statistics were used to compare ZCTAs within high-rate clusters with the rest of the state with respect to measures of rurality/urbanization, demographic composition, economic characteristics, educational attainment, healthcare access, and the food environment.

### Data sources and preparation

#### Hospital discharge, population and ZCTA Data

Hospital discharge data for patients admitted between January 1, 2016 and December 31, 2019 were provided by the Florida Agency for Health Care Administration through a Data Use Agreement with the Florida Department of Health. The following variables were extracted from the dataset: patient age, admission and discharge dates, ZIP code of residence, all International Classification of Diseases, Tenth Revision, Clinical Modification (ICD-10-CM) diagnosis codes, and all International Classification of Diseases, Tenth Revision, Procedure Coding System (ICD-10-PCS) procedure codes. Records for patients between the ages of 18 and 100 with diabetes-related hospitalizations were selected for analysis. Patients with diabetes-related hospitalizations were defined as those who met any of the following criteria:

1. Patients with a principal diagnosis code included in one of the following Agency for Healthcare Research and Quality (AHRQ) Prevention Quality Indicators (PQIs): diabetes with short-term complications (PQI 01), diabetes with long-term complications (PQI 03), and uncontrolled diabetes (PQI 14) [27–29].
2. Patients with a diagnosis of Type 1 or Type 2 diabetes mellitus (ICD-10-CM codes E10.x or E11.x) in any diagnosis field and a procedure code for lower-extremity amputation in any procedure field. These included all diagnosis codes included in AHRQ PQI 16 (lower extremity amputation among patients with diabetes) [30], as well as procedure codes for toe amputations in order to maintain consistency with methods used in the Florida Diabetes Advisory Council Diabetes Report [15].
3. Patients with a diagnosis of Type 1 or Type 2 diabetes mellitus in any diagnosis field, and a principal diagnosis of a complication of diabetes included in the list used to construct the Diabetes Complications Severity Index [31, 32].

The ZIP code to ZCTA crosswalk table developed by John Snow, Inc. was used to join patient address ZIP codes to ZIP code tabulation areas (ZCTAs) [33], and diabetes-related hospitalizations were then aggregated to the ZCTA level. The total number of person-years at risk used to compute ZCTA-level diabetes-related hospitalization rates was obtained by summing the adult populations for each year from 2016 to 2019, which were obtained from the U.S. Census Bureau American Community Survey (ACS) 5-year estimates [23, 34–36]. In order to limit risk of disclosure and minimize bias from areas with low population counts, values for ZCTAs with fewer than 100 adult residents during any year of the study and/or fewer than 10 diabetes-related hospitalizations were excluded from statistical analyses and suppressed in map figures. Therefore, a total of 933 ZCTAs were included for further analysis.

#### Shape files, grocery store, and physician Data

County- and ZCTA-level cartographic boundary shapefiles used for mapping were obtained from the U.S. Census Bureau TIGER Geodatabase and the Florida Geographic Data Library [37, 38]. The number of grocery stores at the ZIP code level was obtained from the 2016 U.S. Census Bureau Annual Economic Survey and joined to ZCTAs [39]. Grocery store density was computed using land area obtained from the ZCTA shapefile. Practice address ZIP codes for physicians with a specialty of family practice or internal medicine who had a clear/active license at the time of data accession (01/2021) were obtained from the Florida Department of Health, Division of Medical Quality Assurance’s Florida Healthcare Practitioner Data Portal [40]. Practice address ZIP codes were joined to ZCTAs and aggregated to the ZCTA level to compute the number of primary care physicians per 10,000 population.

#### Socioeconomic and demographic Data

Socioeconomic and demographic characteristics at the ZCTA level were obtained from ACS 5-year estimates [23, 41–45]. These included: population age distribution and racial/ethnic composition, percent ≥ 25 years of age without a high school education, percent ≥ 25 years of age with a bachelor’s degree or higher, median household income, median household value, percent of families in poverty, percent unemployed, percent without health insurance coverage, and percent of households without a vehicle. Diabetes prevalence estimates at the ZCTA level were obtained from the Centers for Disease Control and Prevention (CDC) Population-Level Analysis and Community Estimates (PLACES) project [21]. Rural-urban classification of ZCTAs was performed using locale assignments published by the National Center for Education Statistics [25].

### Geographic analysis

To adjust for spatial autocorrelation and low populations in some ZCTAs, the ZCTA-level diabetes-related hospitalization rates were smoothed before mapping. The smoothing was performed using the spatial empirical Bayes smoothing approach, employing queen contiguity weights in GeoDa 1.18.0 [46]. This approach shrinks local rate estimates toward the local mean rate within a spatial window containing a given geographic unit and its neighbors [47]. This results in more stable estimates for areas with small populations and large variances, while accounting for the geographic structure of the data [47, 48].

Cluster investigation was performed using Tango and Takahashi’s flexible spatial scan statistic (FSSS), specifying a Poisson model, implemented in FleXScan v3.1.2 [49]. Under the null hypothesis of no clustering, the number of cases in each region in the study area follows a Poisson distribution with mean proportional to the relative number of person-years at risk in the region [49, 50]. To assess whether significant spatial clustering exists, a set of flexibly shaped scanning windows are imposed on each region in the study area. The windows include the individual region as well as all combinations of connected regions up to a specified maximum number of nearest neighbors. A log-likelihood ratio test (LLR) is used to compare the log-likelihood of observing the number of cases that occur within each window with that of the regions outside of it. The regions within the window with the highest value of the LLR comprise the primary cluster. Secondary clusters are identified from the remaining regions in the study area that do not overlap with any cluster with a higher LLR statistic. To increase computational efficiency and avoid including areas that do not have elevated rates in the primary cluster, a restricted LLR statistic was used [50, 51]. This takes the individual rate of each region into account and limits candidates for inclusion in the primary cluster to those with elevated risk at a pre-specified α value. In this study, the maximum scanning window size was specified as 30 ZCTAs, and the default α value of 0.2 was selected for restriction of the likelihood ratio test. Monte Carlo simulation with 999 replications was used for statistical inference. Clusters with LLR test *p*-values ≤ 0.05 and rate ratios (RRs) ≥ 1.5 were reported.

Smoothed estimates of diabetes-related hospitalization rates and cluster identities were imported into QGIS Opensource software, which was used to perform all cartographic manipulations [52]. Jenks’ optimization scheme was used for classification of smoothed diabetes-related hospitalization rate estimates and rate ratios of diabetes-related hospitalization clusters [53].

### Descriptive statistical analyses

Descriptive analyses were performed using SAS version 9.4 [54]. The Shapiro-Wilk test was used to assess normality of distribution of continuous variables. Since all continuous variables were non-normally distributed, median and interquartile range (IQR) were used as measures of central tendency and dispersion, respectively. ZCTAs with missing data for any of the variables assessed were excluded from computations of median and IQR for that variable. Variables with missing data were: median household value (14/933, 1.5%), median household income (4/933, 0.4%), percent of families in poverty (2/933, 0.2%), percent of households without a vehicle (1/933, 0.1%). The Wilcoxon rank sum test was used to assess whether median characteristics of non-cluster ZCTAs differed significantly from those of ZCTAs within clusters of high diabetes-related hospitalization rates. The Chi-square test was used to assess whether the distribution of rural-urban ZCTA locale assignments differed between cluster and non-cluster areas.

## Results

### Distribution of diabetes-related hospitalization rates

**Fig 2:**
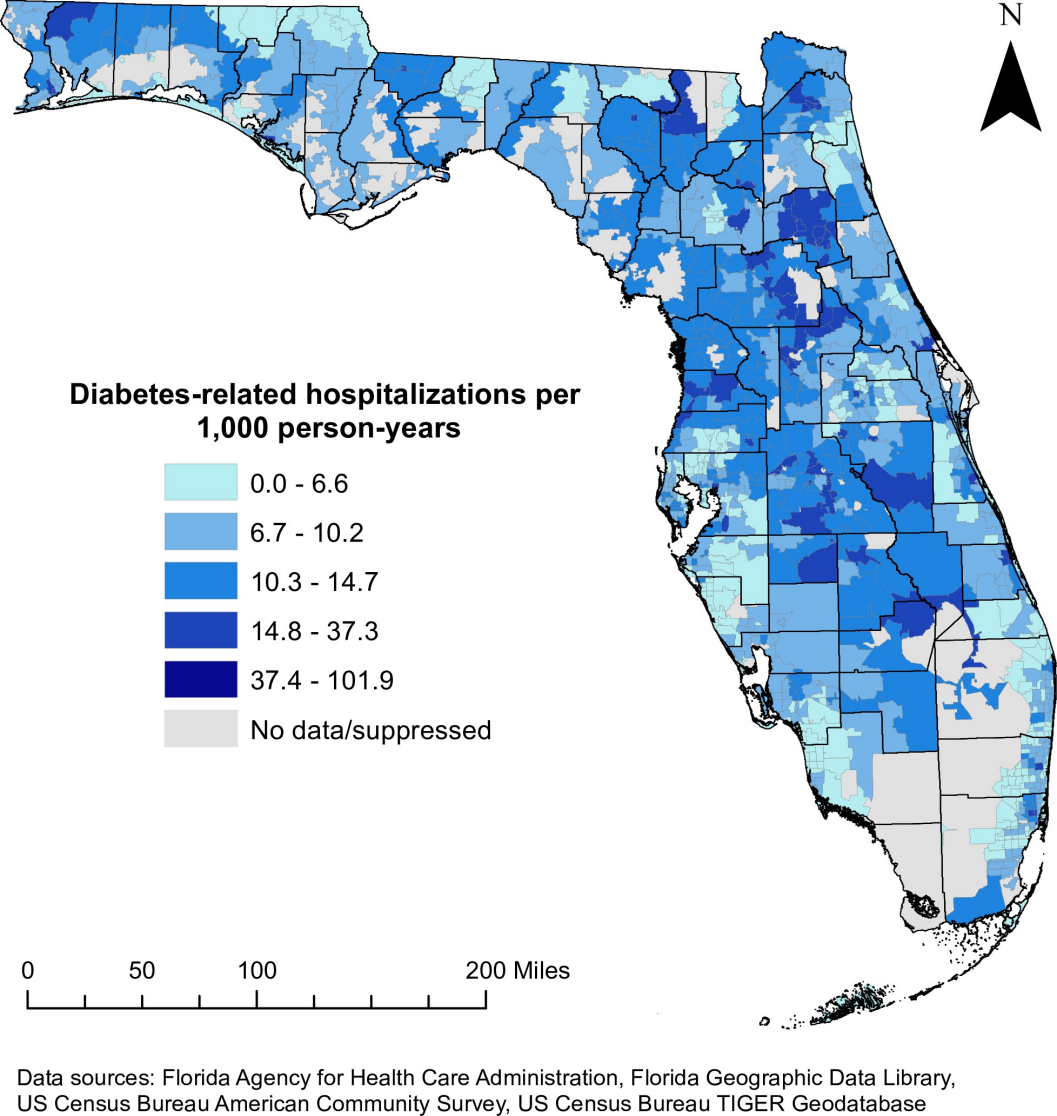
Geographic distribution of spatial empirical Bayes smoothed diabetes-related hospitalization (DRH) rates at the ZIP code tabulation area level in Florida, 2016–2019. Figure is not copyrighted and was created by study authors using non-proprietary open-source software, QGIS.

**Fig 3:**
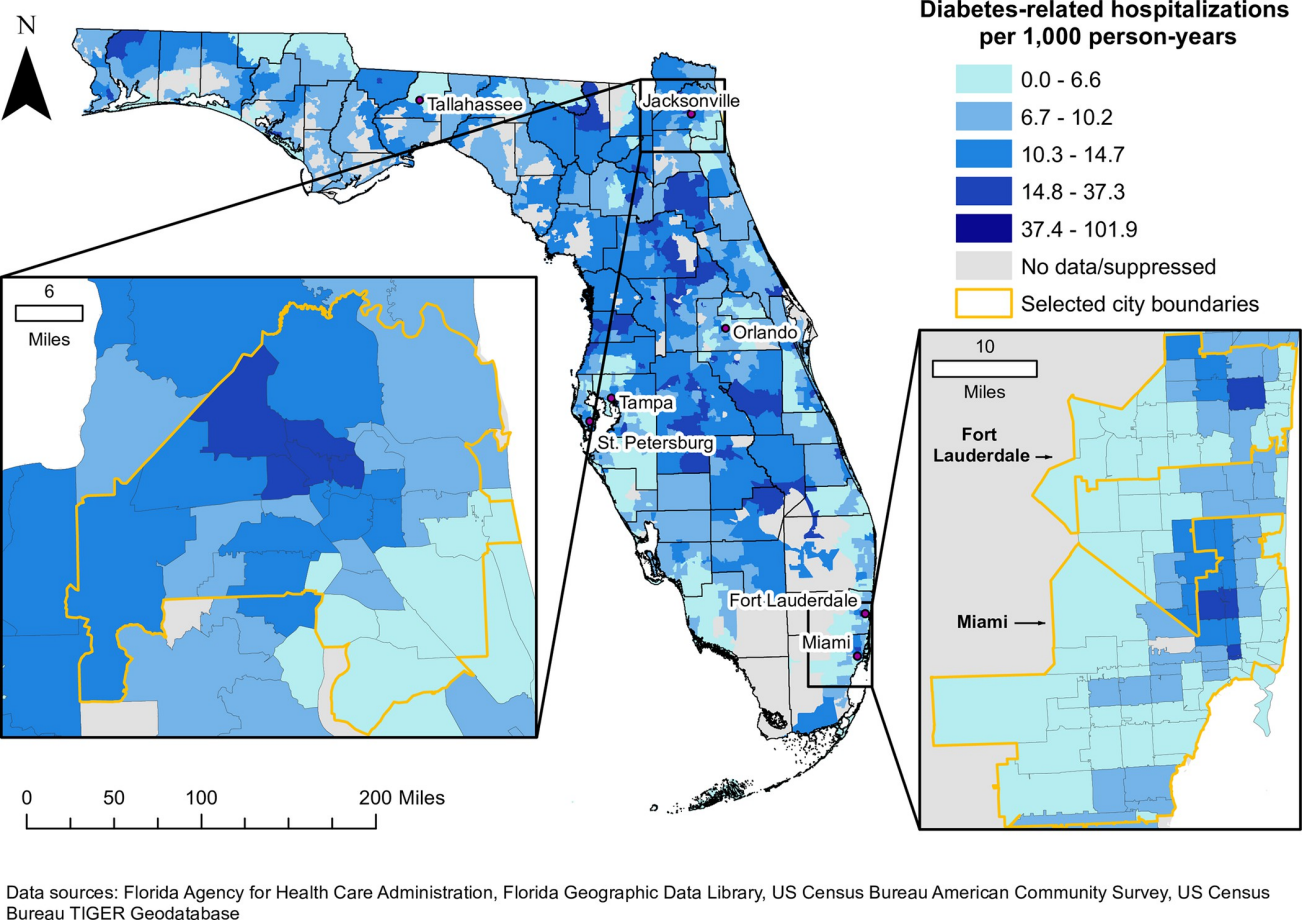
Geographic distribution of spatial empirical Bayes smoothed diabetes-related hospitalization (DRH) rates in selected metropolitan areas in Florida, 2016–2019. Figure is not copyrighted and was created by study authors using non-proprietary open-source software, QGIS.

There was a total of 2,581,031 hospitalizations with a diagnosis of Type 1 or Type 2 diabetes among patients aged 18–100 years. Only 554,133 of these hospitalizations met the inclusion criteria for diabetes-related hospitalizations in this study. The statewide diabetes-related hospitalization (DRH) rate was 8.5 per 1,000 person-years, but spatial empirical Bayes (SEB) smoothed rates at the ZCTA level ranged from 0 to 101.9 (Fig 2). The highest DRH rates tended to occur in ZCTAs in inland central and northeastern Florida, as well as along the Gulf Coast to the north of the Tampa Bay area (Figs 1 and 2). In northwest Florida, ZCTAs to the west of Tallahassee as well as those in the rural northwest panhandle also had relatively high rates of diabetes-related hospitalizations (Figs 1 and 2). While large swaths of ZCTAs with high DRH rates tended to occur in relatively rural parts of the state, the distribution of DRH rates exhibited marked variation within some metropolitan areas (Fig 3). For instance, a group of ZCTAs extending from central Jacksonville to the northern and western fringes of the city was characterized by relatively high DRH rates compared to those in southeastern Jacksonville and closer to the Atlantic Coast. High rates of DRH were also evident in southeastern coastal Florida, an area that consists largely of urban and suburban ZCTAs (Fig 3). Two distinct groups of ZCTAs with high DRH rates were present in this area, one in the city of Fort Lauderdale and the other in northern Miami. Both areas were surrounded by ZCTAs with relatively low DRH rates.

### Significant spatial clusters of high rates of diabetes-related hospitalizations

**Fig 4:**
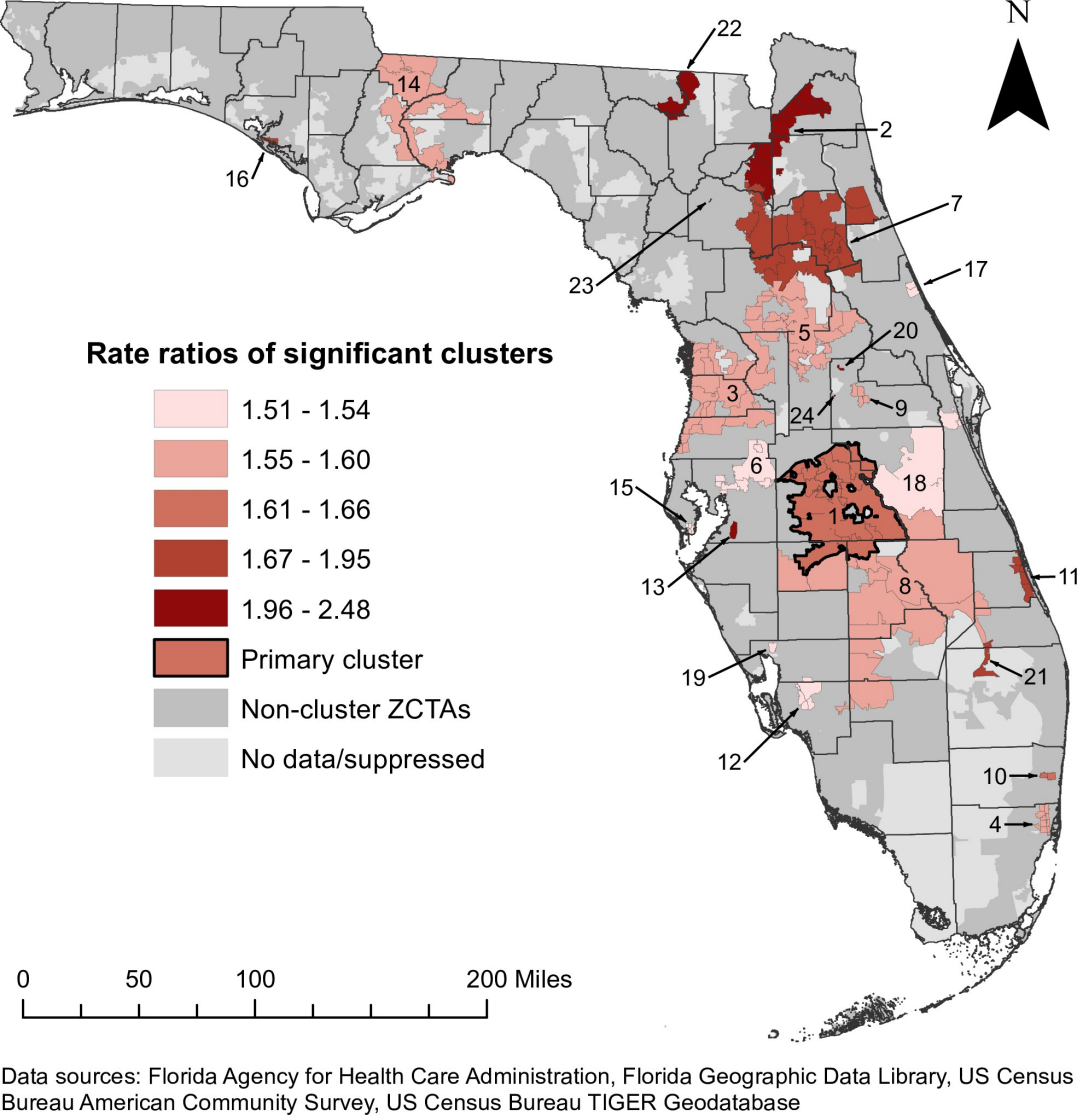
Significant high-rate spatial clusters of diabetes-related hospitalizations at the ZIP code tabulation area level in Florida, 2016–2019. Figure is not copyrighted and was created by study authors using non-proprietary open-source software, QGIS.

Geographic locations and rate ratios of significant high-rate spatial clusters of diabetes-related hospitalizations are displayed in Fig 4. The DRH rate of the primary cluster was 14.0 per 1,000 person-years (Table 1). The rate ratio (RR) of this cluster was 1.65, implying that the rate of diabetes-related hospitalizations within the cluster was 1.65 times that of the state. The primary cluster was also the largest cluster, and was comprised of 24 ZCTAs in central Florida to the east of Tampa and southwest of Orlando (Figs 1 and 4). Most of these ZCTAs were located in Polk County, but the cluster also included portions of Osceola, Highlands, and Hardee Counties (Figs 1 and 4). The majority (79.2%) were classified as fringe or distant rural ZCTAs.

**Table 1:** Significant high-rate spatial clusters of diabetes-related hospitalizations at the ZIP code tabulation area level in Florida, 2016–2019.

Cluster	Location		Size^a^	Number of cases		Rate^b^	RR^c^	*p*
	Region	Counties		Observed	Expected			
1	Central	Polk, Osceola, Highlands, Hardee	24	19915	12099.6	14.0	1.65	0.001
2	Northeast	Duval, Nassau, Baker, Union, Bradford, Clay	11	9029	4222.6	18.2	2.14	0.001
3	East Central	Pasco, Hernando, Citrus, Sumter	21	18349	11620.8	13.4	1.58	0.001
4	Southeast	Miami-Dade	12	16438	10286.3	13.6	1.60	0.001
5	Central	Sumter, Lake, Marion	23	14427	9081.4	13.5	1.59	0.001
6	West Central	Hillsborough, Pasco	11	11227	7390.5	12.9	1.52	0.001
7	Northeast	St. Johns, Putnam, Alachua, Bradford, Marion, Volusia	21	5601	3124.3	15.3	1.79	0.001
8	South Central	Glades, Hendry, Lee, Highlands, Hardee, Okeechobee, St. Lucie, Martin, Osceola	12	6976	4361.9	13.6	1.60	0.001
9	Central	Orange	4	6879	4318.5	13.6	1.59	0.001
10	Southeast	Broward	2	5568	3360.8	14.1	1.66	0.001
11	Southeast	St. Lucie	5	4724	2735.2	14.7	1.73	0.001
12	Southwest	Lee, Charlotte	4	4156	2693.4	13.1	1.54	0.001
13	West Central	Hillsborough	1	1466	706.1	17.7	2.08	0.001
14	Northwest	Gadsden, Leon, Liberty, Wakulla, Franklin	11	3224	2024.7	13.6	1.59	0.001
15	West Central	Pinellas	3	3032	1986.4	13.0	1.53	0.001
16	Northwest	Bay	1	1212	620.2	16.6	1.95	0.001
17	East Central	Volusia	2	2405	1596.3	12.8	1.51	0.001
18	East Central	Osceola, Brevard	4	1733	1126.4	13.1	1.54	0.001
19	Southwest	Charlotte	1	1427	940.9	12.9	1.52	0.001
20	Central	Orange	1	201	81.2	21.1	2.48	0.001
21	Southeast	Palm Beach, Martin	2	398	217.4	15.6	1.83	0.001
22	Northeast	Columbia, Hamilton, Suwannee	1	127	62.8	17.2	2.02	0.001
23	Northeast	Alachua	1	65	26.7	20.7	2.44	0.002
24	Central	Orange	1	56	22.9	20.9	2.45	0.006

^a^Number of ZIP code tabulation areas

^b^Diabetes-related hospitalization rate per 1,000 person-years

^c^Rate ratio

In addition to the primary cluster, 23 secondary clusters were identified, with rate ratios ranging from 1.51 to 2.48 (Table 1 and Fig 4). A total of 179 ZCTAs (19.2% of the 933 ZCTAs included in the analysis) were included in high-rate clusters. The primary cluster in central Florida was bordered by several secondary clusters that had rate ratios ranging from 1.55 to 1.60 (Table 1 and Fig 4). The large secondary cluster to the south of the primary cluster encompassed the majority of Highlands and Okeechobee Counties, and included rural ZCTAs in several other counties in inland south-central Florida (Figs 1 and 4).

**Fig 5:**
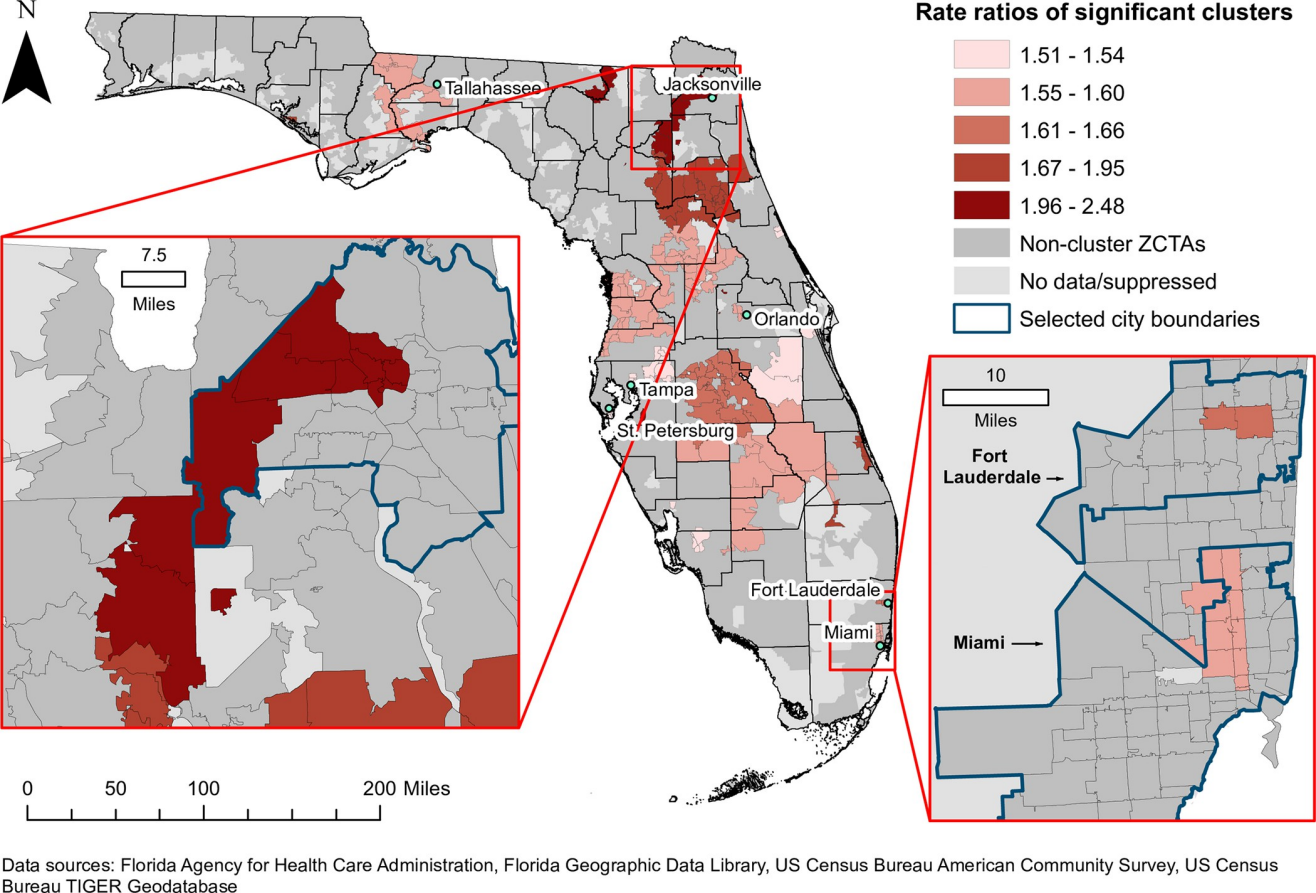
Significant high-rate spatial clusters of diabetes-related hospitalizations in selected metropolitan areas in Florida, 2016–2019. Figure is not copyrighted and was created by study authors using non-proprietary open-source software, QGIS.

There were several large clusters to the north of the primary cluster, spanning a mostly rural area that extended northeast from west central Florida (Fig 4). Cluster 2 had a rate ratio of 2.14, and was composed of 11 ZCTAs, including 6 “large city” ZCTAs in Jacksonville, the most populous city in Florida (Table 1; Figs 4 and 5). The Jacksonville area cluster was relatively large and had one of the highest rate ratios identified in this study. However, high-rate clusters were also identified in several other metropolitan areas in Florida. In southeastern coastal Florida, high-rate clusters were identified in Miami (RR = 1.60) and Fort Lauderdale (RR = 1.66) (Table 1 and Fig 5). Tampa, St. Petersburg, and Orlando were among the other cities that contained ZCTAs in high-rate clusters (Figs 4 and 5). In addition, there was a relatively large cluster (Cluster 14) with a rate ratio of 1.59 that extended west from southern Tallahassee, the state’s capital (Table 1; Figs 4 and 5).

### Characteristics of cluster vs. non-cluster ZCTAs

The median diabetes prevalence estimate for ZCTAs within high-rate clusters of diabetes-related hospitalizations was 15.1% (95% confidence interval [CI]: 14.1, 16.0), with an interquartile range of 13.8 (95% CI: 13.2, 14.5) to 16.7 (95% CI: 15.6, 17.7), which was significantly higher than that of ZCTAs outside of high-rate clusters (11.9% (95% CI: 10.9, 12.9), IQR 10.2 (95% CI: 9.4, 11.0) to 13.5 (95% CI: 12.6, 14.3), *p* < 0.0001) (Table 2). The characteristics of ZCTAs in high-rate clusters differed significantly (*p* < 0.0001) from those that were not in clusters with respect to their level of rurality/urbanization. High-rate clusters had a higher percentage of rural ZCTAs (60.9%) than non-cluster ZCTAs (41.8%). The majority of ZCTAs in non-cluster areas were either classified as suburbs/towns (38.7%) or cities (19.5%), while 24.0% of the ZCTAs within high-rate clusters were suburbs or towns and 15.1% were cities.

**Table 2:** Comparison of characteristics of ZIP code tabulation areas within and outside of clusters of diabetes-related hospitalizations in Florida, 2016–2019.

Variable	Median (interquartile range)		*p* *
	Cluster ZCTAs^a^	Non-cluster ZCTAs^a^	
Diabetes prevalence estimate (percent)^1^	15.1 (13.8–16.7)	11.9 (10.2–13.5)	< 0.0001
*Rurality/urbanization* ^2^ **			
Rural	60.9 (109)	41.8 (315)	< 0.0001
Suburban/town	24.0 (43)	38.7 (292)	
City	15.1 (27)	19.5 (147)	
*Demographic characteristics* ^3^			
Percent non-Hispanic Black	13.7 (4.4–38.4)	7.4 (2.8–15.2)	< 0.0001
Percent Hispanic	10.6 (5.1–20.8)	10.4 (5.1–24.3)	0.3409
Percent 65 years of age and older	20.8 (15.2–27.8)	18.9 (13.8–26.8)	0.0567
Median age	42.4 (36.8–51.1)	42.6 (38.0–50.3)	0.4720
*Economic characteristics* ^3^			
Median household income	$42,060 ($36,551 –$48,214)	$57,816 ($48,153–72,159)	< 0.0001
Percent of families in poverty	14.0 (9.7–19.4)	7.9 (3.2–11.8)	< 0.0001
Percent unemployed	7.3 (5.4–9.0)	4.9 (3.7–6.4)	< 0.0001
Percent of households without a vehicle	6.4 (4.3–11.1)	4.2 (2.5–6.9)	< 0.0001
Median household value	$115,800 ($95,400–154,200)	$215,650 ($156,600 –$294,100)	< 0.0001
*Educational attainment* ^3^			
Percent ≥25 years of age without a high school education	16.7 (12.2–21.2)	9.1 (5.6–14.6)	< 0.0001
Percent ≥25 years of age with a bachelor’s degree or higher	15.3 (11.9–19.2)	28.5 (19.6–41.3)	< 0.0001
*Healthcare access*			
Percent without health insurance coverage^3^	13.9 (10.9–17.8)	11.0 (8.1–15.0)	< 0.0001
Primary care physicians per 10,000 population^4^	3.3 (0.5–10.0)	7.3 (2.6–14.3)	< 0.0001
*Food environment* ^5^			
Grocery stores per 100 square kilometers	1.8 (0–15.4)	6.4 (0.6–23.0)	0.0005

^a^ZIP code tabulation areas

^1^Data source: CDC PLACES

^2^Data source: National Center for Education Statistics

^3^Data source: ACS 2015–2019 5-year estimates

*p-value for Chi-square test (categorical variable) or Wilcoxon rank sum test (continuous variables)

**Percent and frequency presented for categorical variable

Populations of cluster and non-cluster ZCTAs also exhibited significant differences with respect to racial composition, economic indicators, educational attainment, and healthcare and food access (Table 2). The median percentage of non-Hispanic Black residents was significantly (*p* < 0.0001) higher within cluster ZCTAs (13.7%) than non-cluster ZCTAs (7.4%). Populations of ZCTAs within high-rate clusters had significantly (*p* < 0.0001) lower median household income ($42,060) than those of non-cluster ZCTAs ($57,816). In addition, the median percentage of families in poverty was significantly (*p* < 0.0001) higher in cluster ZCTAs (14.0%) than non-cluster ZCTAs (7.9%). The median unemployment rate and percentage of households without access to a vehicle were also significantly (*p* < 0.0001) higher for populations of cluster ZCTAs than those of non-cluster ZCTAs.

Compared to non-cluster ZCTAs, populations of ZCTAs within high-rate clusters tended to have lower levels of educational attainment. The median percentage of adults aged 25 and older without a high school education was significantly (*p* < 0.0001) higher within cluster ZCTAs (16.7%) than non-cluster ZCTAs (9.1%). In addition, the median percentage of adults with a bachelor’s degree or higher was significantly (*p* < 0.0001) lower within cluster ZCTAs (15.3%) than non-cluster ZCTAs (28.5%). The median percentage of the population without health insurance coverage was significantly (*p* < 0.0001) higher within cluster ZCTAs (13.9%) than non-cluster ZCTAs (11.0%). Furthermore, the median number of primary care physicians per capita was significantly (*p* < 0.0001) lower in cluster ZCTAs (3.3 per 10,000 population) than non-cluster ZCTAs (7.3 per 10,000 population). Finally, median density of grocery stores was also significantly (*p* = 0.0005) lower in cluster ZCTAs (1.8 per 100 km^2^) than in non-cluster ZCTAs (6.4 per 100 km^2^).

## Discussion

This study identified significant high-rate spatial clusters of diabetes-related hospitalizations (DRH) at the ZCTA level in Florida, and provides evidence of geographic disparities of DRH rates within the state. Diabetes-related hospitalization rates provide a useful measure of diabetes outcomes at the local level, since statewide surveys such as the Behavioral Risk Factor Surveillance System are not spatially representative at the sub-county level in Florida. There was wide variation in ZCTA-level DRH rates within the study area, with some clusters having rates that were more than double that of the state overall. In addition, the identified spatial clusters exhibited significant differences from the remainder of the state with respect to population demographic and socioeconomic characteristics, level of rurality, and food environment and healthcare resources. Interventions that aim to alleviate the excess burden of DRH in the hotspots identified in this study have the potential to reduce the observed inequities.

Previous research in Florida identified county-level geographic disparities in pre-diabetes and diabetes prevalence [17–20]. The locations of those identified high-prevalence diabetes clusters and the high-rate DRH clusters identified in this study exhibited some overlap, particularly in south-central Florida and near Tallahassee [18–20]. Populations of cluster ZCTAs also tended to have higher diabetes prevalence than those of non-cluster ZCTAs, suggesting that the observed disparities in hospitalization rates may, in part, reflect the burden of diabetes in the population. However, differences between the distribution of DRH rates and diabetes prevalence were also apparent in some parts of the state. This could be indicative of disparities in diabetes care and management. This is consistent with findings of previous research in the US, which identified regional differences in diabetes outcomes and receipt of recommended care [55, 56]. For instance, compared to patients with diabetes from other parts of the country, those from the Southern US are less likely to receive HbA1c testing, are more likely to report foregoing medical care due to cost, and are more likely to experience hypoglycemia [55, 56].

The identified local clusters in this study are important for guiding targeted efforts to improve glycemic control and cardiovascular risk factor management among patients with diabetes in communities with high DRH rates. Exploring characteristics of clusters and their populations can also inform needs-based planning to improve outcomes in these communities. For instance, the largest high-rate DRH clusters were primarily located in rural parts of Florida, consistent with findings of other US studies that have reported rural-urban disparities in diabetes outcomes [57, 58]. This could, in part, reflect a higher burden of diabetes and risk factors such as overweight/obesity and tobacco use among rural populations compared to those of urban areas [59–63]. High DRH rates in rural areas may also reflect challenges to obtaining outpatient care and effective diabetes self-management for rural patients. For example, limited public transportation in rural areas could pose a barrier to attending appointments or obtaining medications in a timely manner. This challenge may be exacerbated by other resource limitations of ZCTAs within high-rate clusters. For instance, cluster ZCTAs tended to have greater distances between grocery stores (which often contain pharmacies), fewer primary care physicians per capita, and populations with more limited vehicle access than the rest of the state. Previous research in Florida reported a negative association between the percentage of rural residents and county-level rates of participation in diabetes self-management education (DSME) programs [19]. In addition, nationwide studies have reported that patients with diabetes in rural areas are less likely to receive routine HbA1c testing, foot and eye examinations than those in metropolitan areas [59, 64].

While high-rate clusters tended to occur in rural rather than urban areas, it is worth noting that the distribution of DRH rates within some of the major cities in Florida varied significantly, and high-rate clusters were identified in several metropolitan areas. Some studies conducted in other states have also reported geographic disparities within urban areas. Examples include hotspots of diabetes-related emergency department visits in central Los Angeles County in California [65], and diabetes-specific inpatient hospitalizations and emergency department visits in New York City [16]. In this study, one of the most notable high-rate clusters occurring in a metropolitan area included part of Jacksonville, located in Duval County in northeastern Florida. An analysis of 2007 data also reported sub-county level disparities in diabetes prevalence, emergency department visits, hospitalizations, and deaths in Duval County [22]. Compared to the rest of the county, residents of central Jacksonville had higher burdens of all the outcomes investigated in that study [22]. The current study found that these central ZCTAs also had relatively high hospitalization rates between 2016 and 2019, and were part of a significant spatial cluster. This suggests that there are persistent geographic disparities with respect to diabetes complications at the sub-county level, and highlights the value of ongoing data collection and analysis at the local level to inform resource allocation.

ZCTAs located in high-rate clusters of diabetes-related hospitalizations tended to have higher proportions of non-Hispanic Black residents compared to the rest of the state. This is consistent with findings of the aforementioned Duval County study [22]. The proportion of non-Hispanic Black residents has also been reported to be a predictor of county-level pre-diabetes [17] and diabetes prevalence in statewide investigations in Florida [20]. Racial disparities with respect to diabetes-related quality of care and management have been reported in numerous studies in the United States, and may contribute to disparities in adverse outcomes [55, 64, 66–68]. Black patients with diabetes have lower odds of receiving HbA1c testing, blood pressure testing, and foot examination at recommended intervals, are less likely to have blood pressure and HbA1c control, and are more than twice as likely to have diabetes-related hospital admissions initiated in the emergency department compared to White patients [55, 64, 66–68]. A Georgia study reported that non-Hispanic Black patients with diabetes had significantly higher rates of hospital discharges and longer hospital stays than non-Hispanic White patients [69].

High-rate DRH clusters were characterized by relative socioeconomic disadvantage when compared to the rest of the state, with populations of cluster ZCTAs having lower median income and educational attainment in addition to higher levels of poverty and unemployment than those of non-cluster ZCTAs. Socioeconomic position can impact diabetes management through pathways such as health behaviors and health literacy, social support, stress, competing demands, and area-level structural factors such as built environment resources [70]. Cost barriers may contribute to medication underuse and foregone medical care among patients with diabetes [56, 71, 72]. Populations of high-rate DRH clusters in this study also tended to have lower levels of health insurance coverage compared to those of non-cluster ZCTAs. Lack of insurance coverage may result in delayed detection of diabetes and initiation of appropriate treatment [73]. Furthermore, patients with diabetes who do not have health insurance coverage are less likely to receive diagnostic testing and examinations at recommended intervals compared to those with private insurance [55, 64, 67, 68].

It is important to note that this study did not distinguish between Type 1 and Type 2 diabetes when computing rates of diabetes-related hospitalizations. However, while Type 1 and Type 2 diabetes differ with respect to risk factors, age of onset, and treatment approaches [2], both are considered ambulatory care sensitive conditions because effective management may prevent or delay hospitalizations due to their complications [27–30, 74]. Furthermore, socioeconomic and/or racial disparities in diabetes technology use, glycemic control, complications, and mortality have been reported among adults with Type 1 diabetes [75–82]. Further research investigating the distribution of hospitalization rates for Type 1 and Type 2 diabetes separately could help elucidate whether the local distribution of DRH rates differs for the two conditions, providing further insights to inform targeted interventions. However, inadequate sample size would preclude investigation of Type 1 diabetes outcomes at the geographic scale used in the present study, since the condition is estimated to represent approximately 5% of diagnosed diabetes cases among U.S. adults [83].

### Strengths and limitations

To our knowledge, this is the first statewide study investigating geographic disparities of diabetes-related hospitalization rates at the sub-county level in Florida. Understanding these disparities is particularly relevant to public health in Florida, where geographic disparities in diabetes prevalence have persisted over the past decade, and substantial increases in diabetes-related hospitalizations, amputations, and hospital costs have been reported [15, 18–20].

This study was conducted using Tango’s flexible spatial scan statistic (FSSS), a robust technique for the detection of high risk/rate spatial clusters [49]. Tango’s FSSS can be used to detect either circular or irregularly shaped clusters and does not suffer from problems of multiple comparisons or pre-selection bias, which are drawbacks of some other cluster detection methods. In addition, the use of hospital discharge data over a four-year period in this study provided a sufficient sample size for investigating the distribution of diabetes-related hospitalization rates at a relatively small geographic unit of analysis (ZIP code tabulation areas). Since this analysis was conducted at a relatively fine geographic scale, it permitted the assessment of spatial patterns that would have been masked with a larger unit of analysis. The observed spatial patterns and cluster locations provide actionable information for public health personnel and community-based organizations, and can help guide the implementation of focused intervention strategies to improve access to care and diabetes management.

However, this study was not without limitations. Direct estimates of diabetes prevalence are not available at the ZCTA level, so diabetes-related hospitalization rates presented in this study are not adjusted for the burden of diabetes in the population. Additionally, this study does not account for patients whose illness warrant hospitalization but do not receive it, for reasons such as travel time to the nearest hospital, or declining care due to cost of hospitalization. While previous research has investigated hospital proximity as a predictor of healthcare utilization for ACSCs, the results have been inconsistent. Findings of some studies suggest that living closer to the hospital increases the likelihood of admission for potentially preventable conditions [84, 85], while others have found no such association [86, 87]. One study in Scotland found that distance from the hospital was a significant predictor of ACSC emergency admissions for certain conditions, but this relationship was not statistically significant for admissions due to complications of diabetes [88]. Finally, this investigation was limited to the state of Florida, and the findings of descriptive analyses comparing cluster and non-cluster ZCTAs may not generalize to other areas. However, the techniques used in the present study can be employed in other states to identify and describe high-rate clusters of diabetes-related hospitalizations and other potentially preventable hospitalizations. Despite the above limitations, the findings of this study are valuable for guiding health planning, resource allocation, and future research.

## Conclusions

This study identified geographic disparities of DRH rates at the ZCTA level in Florida. High-rate clusters tended to: have greater proportions of non-Hispanic Black residents, be in rural areas, have relatively fewer grocery stores and primary care physicians, and have populations with lower income, educational attainment, and employment rates compared to the rest of the state. The identification of high-rate DRH clusters in this study provides useful information to guide resource allocation such that communities with the highest burdens of these potentially preventable hospitalizations are prioritized to reduce the observed disparities. Future research will investigate determinants of hospitalization rates to inform public health planning, resource allocation and interventions.
